# A Longitudinal Investigation of the Relationship between Posttraumatic Stress Symptoms and Posttraumatic Growth in a Cohort of Israeli Jews and Palestinians during Ongoing Violence

**DOI:** 10.1371/journal.pone.0124782

**Published:** 2015-04-24

**Authors:** Brian J. Hall, Leia Y. Saltzman, Daphna Canetti, Stevan E. Hobfoll

**Affiliations:** 1 Department of Psychology, The University of Macau, Macau (SAR), People’s Republic of China; 2 Department of Health, Behavior and Society, Johns Hopkins Bloomberg School of Public Health, Baltimore, Maryland, United States of America; 3 School of Social Work, Boston College, Boston, Massachusetts, United States of America; 4 School of Political Science, University of Haifa, Haifa, Israel; 5 Department of Behavioral Sciences, Rush University Medical Center, Chicago, Illinois, United States of America; Univ of Toledo, UNITED STATES

## Abstract

**Objectives:**

Meta-analytic evidence based on cross-sectional investigations between posttraumatic growth (PTG) and posttraumatic stress disorder (PTSD) demonstrates that the two concepts are positively related and that ethnic minorities report greater PTG. Few longitudinal studies have quantified this relationship so the evidence is limited regarding the potential benefit PTG may have on post-traumatic adjustment and whether differences between ethnic groups exist.

**Methods:**

The current study attempts to fill a substantial gap in the literature by exploring the relationship between PTG and PTSD symptom clusters longitudinally using a nationally representative cohort of 1613 Israelis and Palestinian Citizens of Israel (PCI) interviewed via telephone on three measurement occasions during one year. Latent cross-lagged structural models estimated the relationship between PTG and each PTSD symptom cluster, derived from confirmatory factor analysis, representing latent and statistically invariant PTSD symptom factors, best representing PTSD for both ethnic groups.

**Results:**

PTG was not associated with less PTSD symptom severity in any of the four PTSD clusters, for Jews and PCI. In contrast, PTSD symptom severity assessed earlier was related to later reported PTG in both groups.

**Conclusions:**

This study demonstrates that PTSD symptoms contribute to greater reported PTG, but that PTG does not provide a salutatory benefit by reducing symptoms of PTSD.

## Introduction

The *Al Aqsa Intifada*, a period of conflict in Israel lasting close to five years, produced an environment in which high numbers of civilians were repeatedly exposed to bombings and terror attacks. Bleich, Gelkopf, and Solomon [1] found that nearly half of a nationally representative sample of Israelis reported direct, or indirect (through family member or friend), exposure to terrorism. The high levels of exposure among the general population highlighted the clinical significance of understanding the nuances in the association between mental health outcomes and repeated, and long lasting, exposure to terrorism.

Studies have documented the association between exposure to political violence and terrorism and mental health problems [2–4]. Among the most investigated of these problems is posttraumatic stress disorder (PTSD). Although it is important to evaluate the pathological responses to exposure, it is now well known that relatively few people exposed to terrorism develop PTSD [5], even in populations that face continued exposure and chronic conditions of deprivation [6]. A burgeoning effort has been undertaken to expand Tedeschi and Calhoun’s [7] theoretical work on possible positive adaptations that emerge as a result of coping with potentially traumatic events (PTE), a construct they named posttraumatic growth (PTG).

PTG is broadly defined as a “positive psychological change experienced as a result of the struggle with highly challenging life circumstances” and trauma [8]. PTG does not solely involve a return to pre-trauma levels of functioning (e.g. resiliency [9]), nor does it speak to the capacity to resist developing psychological disorders [10]. Rather, it is defined as achieving an enhanced level of functioning, sense of meaning or spirituality, and developing closer relationships with others that exceed pre-trauma functioning. In order for growth to occur, Tedeschi and Calhoun [7] posited that some level of distress would be necessary to bring about cognitive processes needed to facilitate growth. To what extent psychological distress is related to PTG overtime, and whether PTG may lead to reductions in psychological distress has not been widely explored in the literature

### PTG and Posttraumatic Stress Disorder

The relationship between PTG and psychological distress is one of the most widely debated issues in the PTG literature. In their early theoretical contributions, Tedeschi and Calhoun [7] suggested that an individual can report growth and psychological distress *at the same time*, and that these two dimensions are *unrelated* to each other. However, few studies support the viewpoint that PTG and psychological distress are unrelated [11, 12].

Cross-sectional studies have demonstrated inconsistent empirical findings regarding the relationship between PTG and PTSD symptom severity. Several studies suggest that PTG is positively associated with PTSD symptom severity such that greater PTG is related to greater PTSD symptom severity [10, 13–15]. While others have demonstrated that PTG is associated negatively with PTSD symptom severity such that more PTG is related to less PTSD symptom severity [16, 17].

A recent meta-analysis of 42 studies (N = 11,469) provided greater clarity on this association and demonstrated a significant positive relationship between PTSD and PTG (*r* = .315) [18]. However, the cross-sectional nature of these studies limits the utility of these findings, and fails to solidify the temporal nature and quality (i.e., positive or negative) of the relationship between PTSD symptom severity and PTG. Longitudinal studies can elucidate these relationships further, and allow for testing of several potential temporal associations such that: 1) PTG leads to less PTSD symptom severity, 2) PTG leads to greater PTSD symptom severity, 3) PTSD symptom severity leads to greater PTG, 4) PTG and PTSD symptom severity are unrelated, and 5) PTSD symptom severity leads to less PTG, an association that has received less theoretical and empirical support.

Only two published studies examined the relationship between PTG and PTSD over time [19, 20]. These studies supported the 3^rd^ pathway described above, demonstrating that PTSD symptom severity is associated with greater PTG subsequently reported. However, one study did not have measurements of the main study variables available at each time point [19]. Much of the literature has focused on the overall PTSD construct without considering variation between PTG and symptom clusters of PTSD. Meta-analytic findings suggest that intrusive thoughts are most associated with PTG [25], so modeling reexperiencing symptoms separately from other PTSD symptoms utilizing a longitudinal study design may provide further support for a unique association. However, results from a longitudinal study examining the association between PTG and each PTSD symptom clusters among Katrina survivors [20], demonstrated consistent associations between each symptom cluster and PTG over time.

### Multicultural Considerations in Posttraumatic Growth

The population in Israel is comprised of distinct ethno-cultural groups (i.e., Jews and Palestinian Citizens of Israel; PCI), each with their own unique ideology, religion, and ties to the land. It is therefore important to assess whether PTG and PTSD symptom severity are related in the same way for Jews and PCI. In order to study these relationships, a necessary precondition is to establish the factor structure of PTSD within these samples, and to formally assess measurement invariance between these groups. Only one known study has examined the factor structure of PTSD within an Israeli sample of adults, but did not distinguish between Jewish and PCI respondents [21] and only two have examined the factor structure of PTSD within the context of terrorism [21, 22].

The relationship between PTG and psychological distress may emerge differently for ethnic minority groups [4]. Furthermore, the latent concepts of PTG and PTSD may be influenced by culture, and therefore may differ across different ethnic groups [23, 24]. Minority group members in the United States report greater prevalence of PTG, and minority status is a significant moderator of the relationship between PTG and mental health [25]. A meta-analysis showed that when compared to samples with mostly White participants, samples with greater ethnic minority composition experienced less depression and reported greater well-being as their reported PTG increased. Furthermore, PTG was related to less global distress for these samples, and PTG was related to greater global distress for majority White samples. However, these results are not specific to minority group members, and studies are needed that model this association specifically for minorities. In the longitudinal study conducted among Katrina survivors described above [20], participants identifying as non-Hispanic Black were more likely to report PTG, but effect modification by ethnicity was not evaluated. Therefore, modeling minority status as a potential moderator between PTG and PTSD symptom severity, especially in longitudinal studies, is warranted.

### The Present Study

Given only two known studies have examined the relationship between PTG and PTSD symptom severity generally [19, 20] and only one specifically within the context of ongoing exposure to terrorism [19], the present study aims to provide additional information about this association over time. The latent dimensions of PTSD are also evaluated with regard to this over-time association, which presents a more nuanced approach to understanding the association between PTSD and PTG among those living in environments where repeated exposure is likely. Therefore, the purpose of the present study is to evaluate the relationships between PTSD symptom severity and PTG within a longitudinal framework. Confirmatory factor analysis (CFA) was used to first establish best fitting latent models for PTSD and PTG to ensure that the measurement of each latent construct was appropriate for use in the Jewish and PCI populations. Furthermore, ethnicity was examined as a potential moderator of the temporal relationship between PTG and PTSD symptom severity. In light of previous longitudinal research, we predicted that symptom severity across each PTSD symptom cluster would be associated with increased PTG over time [19, 20].

## Method

Procedure and Participants. A nationally representative sample of 1613 Israelis was randomly surveyed. Comprehensive landline telephone lists (98% of all numbers in Israel) from the Israeli telephone company (Bezeq) database were used. Stratification by region ensured population representation for Jews and PCI. Phone interviews were conducted on three measurement occasions (August 17-September 8, 2004; February 22-March 13, 2005; July 31-October 9, 2005) by a survey institute in Israel using a structured questionnaire [26]. All scales were translated and back translated by language experts and were completed by participants in 30–40 minutes. Initial contact was made by a Hebrew speaker; Arabic and Russian speakers were available if individuals did not speak Hebrew and callbacks were arranged within 24 hours if a Russian or Arabic speaker was not immediately available. Fifteen attempts were made to contact an adult at each telephone number. At the onset of the interview, verbal informed consent was obtained due to the anonymous nature of the telephone survey. Consent was recorded by the survey institute and linked to the survey respondent ID. This study and verbal consent method was approved by the institutional review boards of Kent State University, University of Haifa, and Rush University Medical Center and conducted in compliance with institutional review board protocols. The response rate for eligible participants was 57% [15, 27]. This rate compared favorably with other studies conducted on adults following the September 11, terror attacks within the United States [28]. Participation rates between 30% and 70% are only weakly associated with survey bias, and any potential bias in sampling is addressed by examining the representativeness of the obtained sample [29]. There were no statistical differences between the current sample and the 2003 Israeli Census in terms of sex, ethnicity, age, and education. Of the 1613 (1136 Jews; 477 PCI) participants in the first wave of data collection, 840 (52% overall; 54% Jew; 48% PCI) were retained for 6-month follow-up. The majority of these individuals (716 people, 85% overall; 94% Jews; 62% PCI) were retained at 12-month follow-up.

### Measures

Demographic information was obtained regarding participants’ age, sex, income, and educational attainment.

#### Exposure to terrorism

Participants’ level of exposure was assessed using several items designed to gauge the specific nature of their experiences ranging from the death of a family member or friend, to being in the target location within 48 hours prior to the terror attack. One summed score was created for each measurement occasion that represented the total number of exposures to terrorism, the total number of exposures within three months prior the start of the study (measured at baseline), or since the last measurement occasion for 6-month and 12-month follow-up.

#### PTSD

PTSD symptom severity was measured using the posttraumatic stress disorder symptom scale, interview format (PSS-I) [30]. This scale was validated for use in telephone surveys with Jewish and Palestinian populations in Israel [31]. Participants reported on the severity of PTSD symptoms occurring for at least one month relating to experiences involving a terrorist attack. The PSS-I contains 17 items that assess PTSD symptom criteria based on the *DSM-IV-TR* [32], including items representing reexperiencing, avoidance, and hyperarousal symptom clusters. Items were answered on a 4-point scale ranging from 0 *not at all* to 3 *extremely*. Cronbach alpha was. 85 or higher at each study wave.

#### Posttraumatic growth

Four items, rated from 0 *not at all* to 3 *extremely* from the COR-Evaluation [33] were used to assess posttraumatic growth. This brief scale captures growth in the three domains; self-perception, interpersonal relationships, and philosophy of life; and demonstrated adequate psychometric properties (α = .82) in studies previously conducted in Israel [26]. This brief scale has also directly compared to Tedeschi and Calhoun’s [34] Posttraumatic Growth Inventory (PTGI) and was found to be highly correlated in a college student population (*r* = .85) [17].

### Statistical analysis

#### Confirmatory factor analyses

Factor analyses were conducted on the baseline sample to evaluate measurement models for PTG and PTSD to establish whether these constructs were invariant between ethnic groups. The configural, metric and temporal invariance of PTG and PTSD was also evaluated in multigroup longitudinal measurement models. Establishing metric invariance between groups is a necessary precondition for testing multigroup structural models. If model parameters are non-invariant, the structural pathways cannot be compared, as equivalent constructs do not exist in each group.

#### Fully latent cross-lagged panel analyses

Panel analyses were conducted to evaluate 1) the temporal relationship between PTG and PTSD symptoms and 2) if the relationship over time is consistent among Jewish and PCI groups. Structural models examined the association between PTG and each symptom cluster of PTSD separately due to non-convergence resulting from the statistical complexity of the models including simultaneous regressions with fully latent PTSD.

The cross-lagged model paths were evaluated in a multistep nested models procedure starting with the saturated model (i.e., autoregressive paths, cross-lagged paths, and correlated like-item residuals free to vary between groups). Four additional models were specified, each constraining one pathway at equivalence for Jews and PCI. These models were used to evaluate whether a cross-lagged pathway was equal in terms of significance and strength between Jews and PCI.

Analyses were conducted using Full Information Maximum Likelihood (FIML) for missing data (covariance coverage range 25% to 100%—current study), MLR estimation (i.e., robust methods) was used for non-normally distributed data, using Mplus version 5.1. Four goodness-of-fit indices were used to evaluate the adequacy of the CFA and structural models: the comparative fit index (CFI), the Tucker Lewis Index (TLI), the standardized root mean square residual (SRMR), and the root mean square of approximation (RMSEA). Values equal to, or greater than,. 90 for the CFI and TLI, and values lower than. 08 for the RMSEA and SRMR, were considered indicators of good model fit [35]. Nested CFA and cross-lagged models were compared using Sattora-Bentler χ^2^ difference test (S-B Δχ^2^) incorporating the scaling correction factor for non-normal data provided by Mplus, with a *p* value set at. 05.

## Results

### Descriptive and Bivariate Analyses

Table 1 presents the demographic composition of the sample for Jews (*N* = 1136) and PCI (*N* = 477) and presents the means, standard deviations, and percentages of study variables.

**Table 1:** Means, standard deviations of study variables and demographic composition of the sample.

	Jews (*n* = 1136)			PCI (*n* = 477)		
Variable	%	*M* (*SD*)	Range	%	*M* (*SD*)	Range
**Age**		45.38 (16.56)	18–96		33.28 (11.75)	18–85
**Female**	53.0			52.0		
**Elementary School**	3.7			12.6		
**High School/Post high School**	95.9			86.9		
**Single/widowed/divorced**	35.0			34.0		
**Married/cohabitating**	64.0			66.0		
**Below average income**	34.4			65.0		
**At or above average income**	55.2			33.0		
**T1 No exposure reported**	20.1			44.2		
**T1 Exposure to 1 + events**	79.9			55.7		
**T2 No exposure reported**	24.4			24.5		
**T2 Exposure to 1+ events**	25.5			7.3		
**T3 No exposure reported**	1.4			0.6		
**T3 Exposure to 1+ events**	3.4			0.6		
**T1 Reexperiencing**		3.14 (3.54)	0–15		4.28 (3.73)	0–15
**T2 Reexperiencing**		2.72 (3.39)	0–15		3.78 (3.93)	0–15
**T3 Reexperiencing**		2.30 (3.27)	0–15		4.23 (4.07)	0–15
**T1 Avoidance**		2.29 (2.90)	0–6		5.01 (3.37)	0–6
**T2 Avoidance**		2.02 (2.65)	0–6		4.84 (3.64)	0–6
**T3 Avoidance**		1.84 (2.59)	0–6		5.58 (3.38)	0–6
**T1 Emotional Numbing**		1.50 (1.94)	0–15		2.58 (2.24)	0–15
**T2 Emotional Numbing**		1.28 (1.80)	0–15		2.10 (2.13)	0–15
**T3 Emotional Numbing**		1.11 (1.81)	0–15		2.37 (2.24)	0–15
**T1 Hyperarousal**		3.27 (3.52)	0–15		6.57 (3.92)	0–15
**T2 Hyperarousal**		3.30 (3.44)	0–15		6.91 (3.61)	0–15
**T3 Hyperarousal**		3.03 (3.54)	0–15		7.82 (3.77)	0–15
**T1 PTG**		3.33 (3.63)	0–12		5.21 (3.97)	0–12
**T2 PTG**		3.14 (3.39)	0–12		6.26 (4.00)	0–12
**T3 PTG**		2.29 (3.24)	0–12		5.48 (3.97)	0–12

Not all percentages sum to equal 100 due to missing data. T1 = baseline interview. T2 = 6-month follow-up. T3 = 12-month follow-up. PCI = Palestinian Citizen of Israel. PTG = posttraumatic growth.

### Measurement Model for PTSD

The four factor model of PTSD with correlated latent factors provided adequate fit for Jews (*χ* ^*2*^(112, *N* = 1136) = 369.217, CFI = .93, TLI = .91, RMSEA = .045 (.040 -. 050) SRMR = .039) and PCI (*χ* ^*2*^(112, *N* = 477) = 238.120, CFI = .91, TLI = .88, RMSEA = .049 (.040 -. 057) SRMR = .05). Factor loadings for each of the four latent factors were significant, and above. 44 for Jews and PCI, save for numbing symptoms for the PCI sample. Modification indices called for the residual error for numbing items (i.e., feeling of detachment or estrangement from others and restricted range of affect) to be freely estimated. This pathway was introduced in the models given the considerable conceptual overlap of these items and the improvement in model fit.

### Measurement Model for PTG

A single factor model of PTG fit the data well for Jews χ^2^(2, *N* = 1136) = 10.08, CFI = .99, TLI = .96, RMSEA = .06 (.03 -. 09) SRMR = .02, and for PCI χ^2^(2, *N* = 477) = 10.56, CFI = .98, TLI = .92, RMSEA = .09 (.04 -. 15), SRMR = .03. Factor loadings for the four PTG items for Jews and PCI respectively were. 57/.51 for “Feeling that my life has meaning,”. 78/.72 for “Intimacy with one or more family members,”. 75/.75 for “Closer relations with my friends,” and. 62/,72 for “More confidence in my ability to do things.”

### Cross-Lagged Panel Analyses

#### Reexperiencing symptoms and PTG

**Fig 1:**
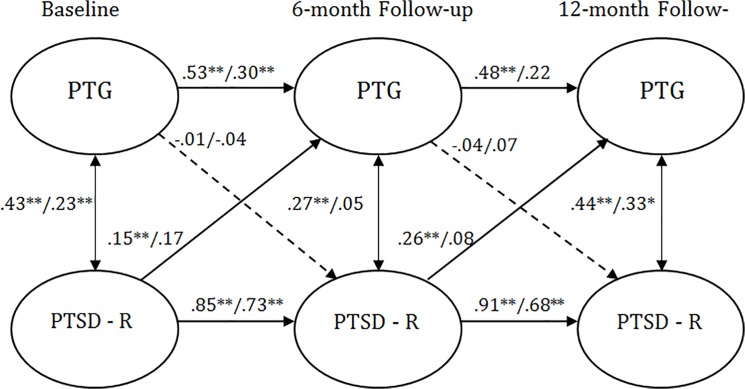
Standardized structural equation modeling results of the saturated fully cross-lagged model demonstrating the temporal relationship between reexperiencing symptoms and posttraumatic growth. All bold cross-lagged model paths are significant for one or both groups in the multigroup models. Dashed lines indicate non-significant path coefficients for both groups. * *p* <. 05. ** *p* <. 01. Values to the left of the diagonal line represent standardized path coefficients for Jews, and values to the right represent standardized path coefficients for PCI. PTG = posttraumatic growth. PTSD—B = posttraumatic stress disorder reexperiencing symptoms.

As can be seen in Table 2, configural, temporal and metric invariance was maintained across the two study groups, which allowed for an evaluation of the cross-lagged models. The path from T1 PTG to T2 reexperiencing was not significant for Jews (β = -0.01) or PCI (β = -0.04). The path from T1 reexperiencing to T2 PTG was significant for Jews (β = .15, *p* = .004) and PCI (β = .17, *p* = .04). The path from T2 PTG to T3 reexperiencing symptoms was not significant for Jews (β = -0.04) or PCI (β = 0.07). Finally, the path from T2 reexperiencing to T3 PTG was significant for Jews (β = 0.26, *p* <. 001) but not for PCI (β = 0.08). Other than this final pathway, no differences were noted between the Jewish and PCI samples. This fully saturated model is depicted by Fig 1.

**Table 2:** Multigroup Cross-lagged Analysis of Reexperiencing Symptoms.

Model	S-B *χ* ^*2*^	*df*	CFI	TLI	RMSEA	SRMR	BIC	S-B Δ*χ* ^*2*^
**Model 1 (configural)**	1001.96	564	.94	.92	.031 (.028 -. 034)	.05	76183.98	—
**Model 2 (temporal invariance)**	1029.93	592	.94	.93	.030 (.027 -. 033)	.05	76015.52	28, 21.41
**Model 3 (metric invariance)**	1056.56	599	.94	.93	.031 (.028 -. 034)	.05	75996.50	35, 42.32
**Model 1 (cross-lagged parameters freed)**	1103.81	607	.93	.92	.03 (.03–0.04)	.06	75992.81	—
**Model 2 (T1 PTG to T2 PTSD-R fixed)**	1103.86	608	.93	.92	.03 (.03–0.04)	.06	75985.54	1,. 03
**Model 3 (T1 PTSD-R to T2 PTG fixed)**	1104.39	608	.93	.92	.03 (.03–0.04)	.06	75985.69	1,. 47
**Model 4 (T2 PTG to T3 PTSD-R fixed)**	1104.67	608	.93	.92	.03 (.03–0.04)	.06	75986.42	1,. 70
**Model 5 (T2 PTSD-R to T3 PTG fixed)**	1105.21	608	.93	.92	.03 (.03–0.04)	.06	75987.08	1, 1.14

S-B *χ* ^*2* =^ Sattora-Bentler *χ* ^*2*^. CFI = comparative fit index. TLI = Tucker Lewis Index. RMSEA = root mean square of approximation. SRMR = standardized root mean square residual. BIC = Bayesian information criterion. S-B Δ*χ* ^*2* =^ Sattora-Bentler *χ* ^*2*^ difference test. PTG = posttraumatic growth. PTSD-R = posttraumatic stress disorder reexperiencing symptoms.

#### Avoidance symptoms and PTG

**Fig 2:**
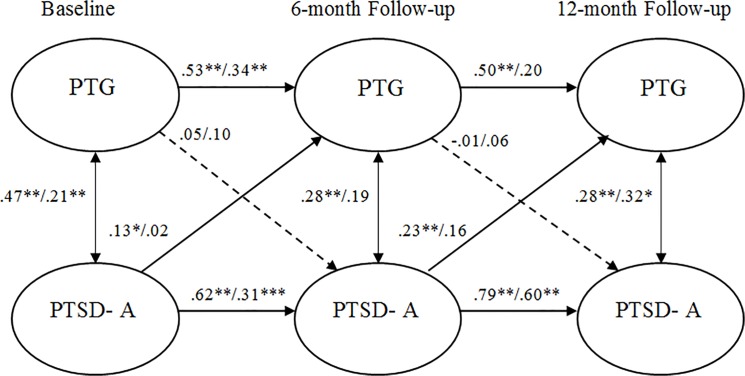
Standardized structural equation modeling results of the saturated fully cross-lagged model demonstrating the temporal relationship between avoidance symptoms and posttraumatic growth. All bold cross-lagged model paths are significant for one or both groups in the multigroup models. Dashed lines indicate non-significant path coefficients for both groups. * *p* <. 05. ** *p* <. 01. Values to the left of the diagonal line represent standardized coefficients for Jews, and values to the right represent standardized path coefficients for PCI. PTG = posttraumatic growth. PTSD—A = posttraumatic stress disorder avoidance symptoms.

As shown in Table 3, configural, temporal and metric invariance was maintained across the two study groups, which allowed for an evaluation of the cross-lagged models. The path from T1 PTG to T2 avoidance symptoms were non-significant Jews (β = 0.05) and PCI (β = 0.10). The path from T1 avoidance to T2 PTG was significant for Jews (β = .13, *p* = .04) but not for PCI (β = .02). The path from T2 PTG to T3 avoidance symptoms was not significant for Jews (β = -0.01) or PCI (β = .06). In the final model, the path from T2 avoidance symptoms to T3 PTG was significant for Jews (β = 0.23, *p* = .002) but not for PCI (β = .16). Despite differences in the significance of model paths, when paths were constrained at equality for both groups, no significant differences were noted. This fully saturated model is depicted by Fig 2.

**Table 3:** Multigroup Cross-lagged Analysis of Behavioral Avoidance Symptoms.

Model	S-B *χ* ^*2*^	*df*	CFI	TLI	RMSEA	SRMR	BIC	S-B Δ*χ* ^*2*^
**Model 1 (configural)**	330.74	204	.97	.96	.028 (.022 -. 033)	.04	54686.91	—
**Model 2 (temporal invariance)**	341.67	220	.97	.92	.026 (.021 -. 031)	.04	54581.44	16, 9.30
**Model 3 (metric invariance)**	364.46	224	.97	.96	.028 (.023 -. 033)	.04	54578.77	20, 28.90
**Model 1 (cross-lagged parameters freed)**	400.64	232	.96	.95	.030 (.025 -. 035)	.05	54561.11	—
**Model 2 (T1 PTG to T2 PTSD-A fixed)**	400.86	233	.96	.95	.030 (.025 -. 035)	.05	54553.94	1,. 19
**Model 3 (T1 PTSD-A to T2 PTG fixed)**	401.80	233	.96	.95	.030 (.025 -. 035)	.05	54554.83	1,. 98
**Model 4 (T2 PTG to T3 PTSD-A fixed)**	400.91	233	.96	.95	.030 (.025 -. 035)	.05	54553.97	1,. 23
**Model 5 (T2 PTSD-A to T3 PTG fixed)**	400.52	233	.96	.95	.030 (.025 -. 035)	.05	54553.91	1,. 08

S-B *χ* ^*2* =^ Sattora-Bentler *χ* ^*2*^. CFI = comparative fit index. TLI = Tucker Lewis Index. RMSEA = root mean square of approximation. SRMR = standardized root mean square residual. BIC = Bayesian information criterion. S-B Δ*χ* ^*2* =^ Sattora-Bentler *χ* ^*2*^ difference test. PTG = posttraumatic growth. PTSD-A = posttraumatic stress disorder avoidance symptoms.

#### Emotional numbing symptoms and PTG

As shown in Table 4, configural, temporal and metric invariance was maintained across the two study groups, which allowed for an evaluation of the cross-lagged models. The path from T1 PTG to T2 numbing symptoms Jews was (β = -0.10, *p* = .07) and PCI (β = -0.04, *p* = .67). T1 numbing to T2 PTG Jews was (β = .07, *p* = .25) and PCI (β = .29, *p* = .001). The path was significantly different between these groups.

**Table 4:** Multigroup Cross-lagged Analysis of Emotional Numbing Symptoms.

Model	S-B *χ* ^*2*^	*df*	CFI	TLI	RMSEA	SRMR	BIC	S-B Δ*χ* ^*2*^
**Model 1 (configural)**	839.17	558	.95	.94	.025 (.021 -.028)	.05	75694.18	—
**Model 2 (temporal invariance)**	863.44	586	.95	.94	.025 (.021 -. 028)	.05	75522.56	28, 18.41
**Model 3 (metric invariance)**	888.92	593	.95	.94	.025 (.021 -. 028)	.05	75500.86	35, 37.80
**Model 1 (cross-lagged parameters freed)**	923.37	601	.94	.93	.026 (.022 -. 029)	.06	75480.69	—
**Model 2 (T1 PTG to T2 PTSD-N fixed)**	923.54	602	.94	.93	.026 (.022 -. 029)	.06	75473.52	1,. 15
**Model 3 (T1 PTSD-N to T2 PTG fixed)**	928.57	602	.94	.93	.026 (.023 -. 029)	.06	75478.74	1, 4.56*
**Model 4 (T2 PTG to T3 PTSD-N fixed)**	924.85	602	.94	.93	.026 (.023 -. 029)	.06	75475.05	1, 1.30
**Model 5 (T2 PTSD-N to T3 PTG fixed)**	924.07	602	.94	.93	.026 (.022 -. 029)	.06	75474.18	1,. 61

S-B *χ* ^*2* =^ Sattora-Bentler *χ* ^*2*^. CFI = comparative fit index. TLI = Tucker Lewis Index. RMSEA = root mean square of approximation. SRMR = standardized root mean square residual. BIC = Bayesian information criterion. S-B Δ*χ* ^*2* =^ Sattora-Bentler *χ* ^*2*^ difference test. PTG = posttraumatic growth. PTSD-N = posttraumatic stress disorder numbing symptoms.

*p <. 05.

**p <. 001.

**Fig 3:**
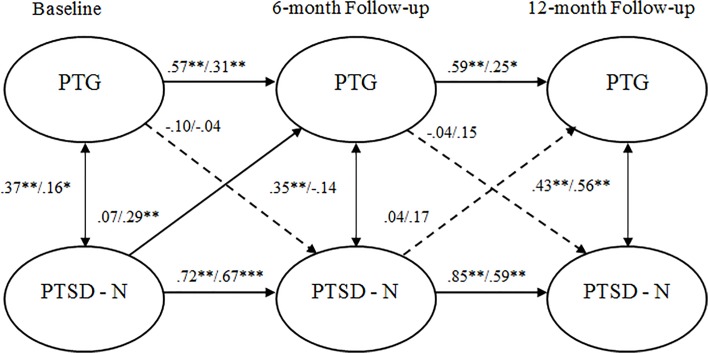
Standardized structural equation modeling results of the saturated fully cross-lagged model demonstrating the temporal relationship between numbing symptoms and posttraumatic growth. All bold cross-lagged model paths are significant for one or both groups in the multigroup models. Dashed lines indicate non-significant path coefficients for both groups. * *p* <. 05. ** *p* <. 01. Values to the left of the diagonal line represent standardized coefficients for Jews, and values to the right represent standardized path coefficients for PCI. PTG = posttraumatic growth. PTSD—N = posttraumatic stress disorder numbing symptoms.

The path from T2 PTG to T3 numbing symptoms for Jews was (β = -.04, *p* = .59) and PCI (β = .15, *p* = .27). In the final model, the path from T2 numbing symptoms to T3 PTG for Jews was (β = 0.04, *p* = .50) and PCI (β = .17, *p* = .19). This fully saturated model is depicted by Fig 3.

#### Hyperarousal symptoms and PTG

As shown in Table 5, configural and temporal invariance was found between the two groups. However, metric invariance was not was upheld and therefore the multigroup cross-lagged model could not be estimated. In order to evaluate the structural relationship between hyperarousal and PTG, cross-lagged models were specified separately for Jews and PCI.

**Table 5:** Multigroup results for cross-lagged analysis of Hyperarousal Symptoms.

Model	S-B *χ* ^*2*^	*df*	CFI	TLI	RMSEA	SRMR	BIC	S-B Δ*χ* ^*2*^
**Model 1 (configural)**	922.85	564	.95	.94	.028 (.025 -. 031)	.05	79288.34	—
**Model 2 (temporal invariance)**	947.40	592	.95	.94	.027 (.024 -. 030)	.05	79114.27	28, 19.55
**Model 3 (metric invariance)**	1014.77	599	.94	.93	.026 (.026 -. 032)	.06	79140.53	35, 73.61**
**Model 1 Jew (all cross-lagged paths free)**	528.22	300	.95	.95	.026 (.022 -. 029)	.05	55219.40	—
**Model 2 Jews (T1 PTSD-H to T2 PTG = 0)**	529.49	301	.95	.95	.026 (.022 -. 029)	.05	55213.98	1,. 99
**Model 3 Jews (T1 PTG to T2 PTSD-H = 0)**	535.28	301	.95	.94	.026 (.023 -. 030)	.05	55220.73	1, 7.22*
**Model 4 Jews (T2 PTSD-H to T3 PTG = 0)**	528.02	301	.95	.95	.026 (.022 -. 029)	.05	55212.95	1,-.11
**Model 5 Jews (T2 PTG to T3 PTSD-H = 0)**	541.24	301	.95	.94	.027 (.023 -. 030)	.06	55228.77	1, 10.18**
**Model 1 PCI (all cross-lagged paths free)**	471.51	300	.90	.89	.029 (.029 -. 040)	.08	23736.90	—
**Model 2 PCI (T1 PTSD-H to T2 PTG = 0)**	473.64	301	.90	.89	.035 (.029 -. 041)	.08	23733.20	1, 1.65
**Model 3 PCI (T1 PTG to T2 PTSD-H = 0)**	474.85	301	.90	.89	.035 (.029 -. 041)	.08	23733.96	1, 3.39
**Model 4 PCI (T2 PTSD-H to T3 PTG = 0)**	471.49	301	.90	.89	.034 (.028 -. 040)	.08	23730.78	1, -0.03
**Model 5 PCI (T2 PTG to T3 PTSD-H = 0)**	474.91	301	.90	.89	.035 (.029 -. 041)	.08	23734.48	1, 2.64

S-B *χ* ^*2* =^ Sattora-Bentler *χ* ^*2*^. CFI = comparative fit index. TLI = Tucker Lewis Index. RMSEA = root mean square of approximation. SRMR = standardized root mean square residual. BIC = Bayesian information criterion. S-B Δ*χ* ^*2* =^ Sattora-Bentler *χ* ^*2*^ difference test. PCI = Palestinian Citizen of Israel. PTG = posttraumatic growth. PTSD-H = posttraumatic stress disorder hyperarousal symptoms.

*p <. 05.

**p <. 001.

#### Hyperarousal and PTG: Jews

In order to test whether the individual cross-lagged paths meaningfully contributed to the model, structural pathways were deleted starting with the T2 variables regressed on T1 variables, and then for the T3 variables regressed on T2 variables.

First, in Model 2, fixing the path from T1 PTG to T2 hyperarousal symptoms to zero yielded a non-significant result, S-B Δ*χ* ^*2*^(1, *N* = 1136) = 0.99. This indicated that this path did not contribute to the model, and that PTG did not predict later hyperarousal symptoms for Jews. The non-significant model path from the fully saturated model was (β = -0.05, *p* = .26).

In model 3, the paths from T1 hyperarousal to T2 PTG were fixed at zero. There was a significant difference between the saturated and constrained models, S-B Δ*χ* ^*2*^(1, *N* = 1136) = 7.22 *p <*. 001. This indicated that this path contributed to the model. The significant model path from the fully saturated model was (β = 0.13, *p* = .004), which demonstrated a small effect size relationship between T1 hyperarousal predicting T2 PTG for Jews.

In model 4, the paths from T2 PTG to T3 hyperarousal was fixed at zero. There were no significant differences between the saturated and constrained models, S-B Δ*χ* ^*2*^(1, *N* = 1136) = -0.11, *p >*. 05. This indicated that this path did not contribute to the model, and that T2 PTG did not predict T3 hyperarousal for Jews. The non-significant model path from the fully saturated model was (β = 0.04, *p* = .57).

**Fig 4:**
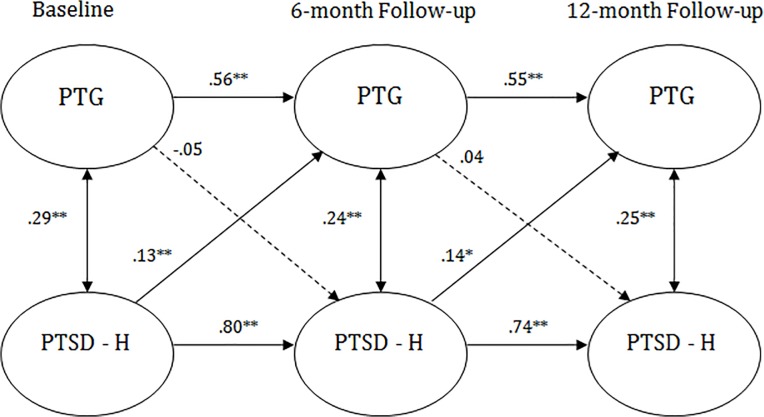
Standardized structural equation modeling results of the saturated fully cross-lagged model demonstrating the temporal relationship between hyperarousal symptoms and posttraumatic growth for the Jewish Sample. All bold cross-lagged model paths are significant for the models. Dashed lines indicate non-significant path coefficients. * *p* <. 05. ** *p* <. 01. PTG = posttraumatic growth. PTSD—H = posttraumatic stress disorder hyperarousal symptoms.

In model 5, the path from T2 hyperarousal to T3 PTG was fixed at zero. Results revealed a significant difference between the saturated and constrained models, S-B Δ*χ* ^*2*^(1, *N* = 1136) = 10.18, *p <*. 001. This indicated that this path contributed to the model. The significant model path from the fully saturated model was (β = 0.14, *p* = .03), which indicated that T2 hyperarousal predicted T3 PTG for Jews. This fully saturated model is depicted by Fig 4.

#### Hyperarousal and PTG: PCIs

**Fig 5:**
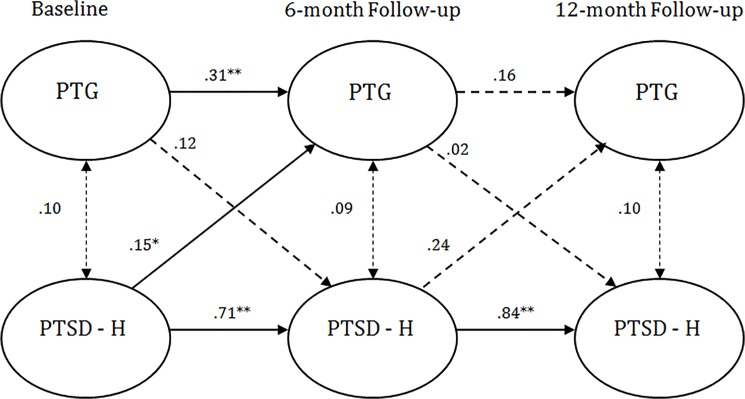
Standardized structural equation modeling results of the saturated fully cross-lagged model demonstrating the temporal relationship between hyperarousal symptoms and posttraumatic growth for the PCI Sample. All bold cross-lagged model paths are significant for the models. Dashed lines indicate non-significant path coefficients. Asterisks indicate significance values for covariances and autoregressive paths. * *p* <. 05. ** *p* <. 01. PTG = posttraumatic growth. PTSD—H = posttraumatic stress disorder hyperarousal symptoms.

The first and most saturated cross-lagged model allowed all cross-lagged structural paths to be freely estimated. Covariances between latent variables at each measurement occasion were also freely estimated. This initial model fit the data well, *χ* ^*2*^(297, *N* = 477) = 471.51, CFI = .90, TLI = .89, RMSEA = .029 (.029 -. 040), SRMR = .08, BIC 23736.90. This fully saturated model is depicted by Fig 5.

In order to test whether the individual cross-lagged paths meaningfully contributed to the model, structural pathways were deleted starting with the T2 variables regressed on T1 variables, and then for the T3 variables regressed on T2 variables. First, in Model 2, fixing the path from T1 PTG to T2 hyperarousal symptoms to zero yielded a non-significant result, S-B Δ*χ* ^*2*^(1, *N* = 477) = 1.65, *p >*. 05. This indicated that this path did not contribute to the model, and that PTG did not predict later hyperarousal symptoms for PCI. The non-significant model path from the fully saturated model was (β = 0.12, *p* = .15).

In model 3, the paths from T1 hyperarousal to T2 PTG were fixed at zero. Results failed to reveal a significant difference between the saturated and constrained models, S-B Δ*χ* ^*2*^(1, *N* = 477) = 3.39. This indicated that this path did not contribute to the model, and that T1 hyperarousal did not predict T2 PTG for PCI. The non-significant model path from the fully saturated model was (β = 0.15, *p* = .05).

In model 4, the paths from T2 PTG to T3 hyperarousal was fixed at zero. Results revealed no significant differences between the saturated and constrained models, S-B Δχ^2^(1, *N* = 477) = -.03. This indicated that this path did not contribute to the model, and that T2 PTG did not predict T3 numbing for PCI. The non-significant model path from the fully saturated model was (β = 0.02, *p* = .83). In model 5, the path from T2 numbing to T3 PTG was fixed at zero. Results revealed no significant differences between the saturated and constrained models, S-B Δχ^2^(1, *N* = 477) = 2.64. This indicated that this path did not contribute to the model, and that T2 hyperarousal did not predict T3 PTG for PCI. The non-significant model path from the fully saturated model was (β = 0.24, *p* = .07).

## Discussion

Utilizing data gathered from a representative sample of Israeli Jews and PCI, the results supported a generally cross-culturally sound representation of PTSD represented by a four-factor correlated model of reexperiencing, avoidance, emotional numbing, and hyperarousal [36]. Importantly, each of these factors, save for hyperarousal, were equivalently measured between Jews and PCI. PTG was not associated with less PTSD symptom severity in any of the four PTSD clusters, for both Jews and PCI. In contrast, PTSD symptom severity was generally predictive of PTG such that PTSD assessed earlier was related to later reported PTG in both groups, calling into question the salutogenic utility of the PTG construct.

### The Best Fitting Model of PTSD and PTG for Jews and PCIs

The results demonstrated that PTSD evidenced adequate cross-cultural generalizability. Three of the four symptom clusters for PTSD were measured equivalently between the Jewish and PCI samples. The symptoms of reexperiencing, avoidance, and emotional numbing remained unchanged over time, and the strength of the factor loadings was invariant across groups. A different picture emerged for the hyperarousal symptom cluster. Although this construct demonstrated temporal stability, the strength of the associations among the factor loadings was heterogeneous across groups. Although the models were not entirely equivalent in terms of the strength of the parameter estimates across groups, the four-factor model was the best fitting among those that were tested.

The PTG construct was equivalently measured between the Jewish and PCI samples. For each group, the four-items loaded onto one latent factor, and this model fit the data reasonably well for both groups. This investigation, although limited by the few items in the scale, provides evidence that the PTG construct was measured adequately within a sample that has experienced ongoing exposure or threat of exposure to terrorism. It also provides evidence that the construct evidenced generalizability between samples and this allowed multigroup analysis to proceed.

### Evaluating the Relationship between PTSD and PTG

The results of the cross-lagged models for PTSD and PTG demonstrated a clear and relatively consistent finding: PTSD symptom cluster severity was associated with greater concurrent and subsequent PTG. At no point was self-reported PTG related to less PTSD symptom cluster severity either cross-sectionally or prospectively. These findings are consistent with previous studies measuring the association between PTSD and PTG [10, 13–15, 18–20] and add to the growing number of studies suggesting that PTG and psychopathology are positively related to one another within terrorism contexts [10, 14, 15, 18, 27].

Specific associations between PTSD symptom clusters and PTG revealed that reexperiencing, avoidance, and hyperarousal symptoms were consistently associated with greater PTG between both measurement periods for the Jewish sample, but between T1 and T2 and between T2 and T3, and at neither time point, respectively, for the PCI. The association between reexperiencing and hyperarousal and PTG supports theory suggesting rumination or cognitive processing is needed for growth to occur [7]. Avoidance symptoms may be related to denial or represent an illusory side of PTG posited by researchers who claim that reported PTG can be truly related to personal growth and also to self-deception [37, 38]. Numbing symptoms were not consistently associated with PTG. This comports well with the theory that cognitive processing is needed for PTG to occur [7].

The effect sizes for the cross-sectional associations when significant were medium [39], and stronger than the cross-lagged effects. This is not surprising but notable, and suggests that cross-sectional studies may exaggerate the relationship between PTSD and PTG. Although this study does not speak directly to causality, these findings are highly suggestive that PTG does not lead to positive mental health outcomes.

### Ethnicity as a Moderator of the Relationship between PTG and PTSD

Within the context of the current study, ethnic minority status, as defined as being a PCI rather than a Jewish Israeli, did not lead to a different general pattern of association between PTG and any of the PTSD symptom clusters. However, the association between PTG and the PTSD symptom clusters was not as strong for the PCI sample compared to the Jewish sample. When significant associations were noted, they only existed between T1 and T2, and not from T2 to T3. The only association that was significant in the PCI sample, and not in the Jewish sample, was between emotional numbing and PTG. For PCI, numbing at T1 was related to greater PTG at T2. Overall, the PCI sample did not evidence a reduction in the severity of any PTSD symptom cluster as a result of self-reported PTG. This finding is consistent with previous research in Israel [10, 14, 15, 27], but contradictory to studies conducted within the United States [25].

Although PTG demonstrated temporal metric invariance between the two samples, PTG was not stable over time for PCI. The autoregressive pathway for PTG between T2 and T3 was not significant in the models. This indicated a lack of stability (i.e., test retest reliability) for this construct between the later follow-up periods. It may be possible that as this group gained temporal distance from the *Al Aqsa Intifada* period, the PTG that they reported began to weaken.

### Strengths and Limitations of the Current Study

This study has several notable strengths. The longitudinal design tested the temporal relationships between PTSD symptom clusters and PTG and this overcomes a serious limitation of the majority of studies testing these associations. A second strength was to evaluate the factor structure of PTSD and PTG and to demonstrate the equivalence of these constructs between different cultural groups. Additionally, the large sample size allowed for ample statistical power to examine the multigroup models reliably. Further, this study extends the literature by examining these processes in a sample that has experienced ongoing and repeated psychological trauma. Taken together, this study contributes significantly to our understanding of how PTSD and PTG are related, the cross-cultural generalizability of PTSD, and the role of ethnic minority status in this relationship.

Despite its strengths, this study also had several limitations. The study design, although longitudinal, cannot discern strict casual relatedness among the study variables since the effects of unmeasured influences cannot be ruled out. In particular, participants’ subjective report of the severity and centrality of their traumatic event exposure, a well documented predictor of mental health outcomes [40–42], was not measured. It may be possible that variation exists in the degree to which terrorism exposure is experienced by our participants as traumatic and may explain variations in the extent to which PTG was expressed [8].

Furthermore, this study captures one discrete period of time characterized by the pressure of ongoing threat of further attacks. As a result, PTG in this context may be different than growth reported following single-incident trauma exposure [10]. In particular, this environment may provide little opportunity of entering a resolution phase where individuals are free from psychopathology, at which point their PTSD symptoms would likely be unrelated to PTG [8].

The limited number of items included in the PTG measure restricted our ability to assess the relationship between PTSD and PTG. Although the items used in this study to measure PTG were found to correlate highly with the PTGI [34], the full dimensionality of the construct was not captured by these four items. As a result, conclusions about the relationship between PTSD and PTG can be made for only three of the five domains of PTG commonly studied. This study is unable to speak to the potential relationship between PTSD and growth in the areas of spirituality and new life possibilities. Furthermore this study did not account for a potential non-linear relationship (quadratic) relationship between PTSD symptom severity and PTG [18].

## Conclusions

Research has documented that the majority of people who experience a major traumatic event, such as terrorism, are resilient [9]. However, the concept of growth reaches beyond resiliency and provides hope for an alternative outcome to PTSD and psychological suffering following trauma. As a result it is essential to understand the relationship between growth and distress over time, and to determine if the relationship varies between majority and minority status populations. This paper contributes to the existing literature by demonstrating that PTSD symptom severity, across symptom clusters and for two ethic groups, contributed to increased self-reported PTG, but that at no time did PTG lead to reduced symptom severity.
